# Microsatellite multiplex assay for the analysis of Atlantic sturgeon populations

**DOI:** 10.1007/s13353-014-0216-y

**Published:** 2014-05-04

**Authors:** H. Panagiotopoulou, D. Popovic, K. Zalewska, P. Weglenski, A. Stankovic

**Affiliations:** 1 Institute of Biochemistry and Biophysics, Polish Academy of Sciences, Pawińskiego 5a, 02-106 Warsaw, Poland; 2 Centre of New Technologies (CeNT), University of Warsaw, ul. Żwirki i Wigury 93, 02-089 Warsaw, Poland; 3 Faculty of Biology, Institute of Genetics and Biotechnology, University of Warsaw, Pawińskiego 5a, 02-106 Warsaw, Poland; 4 The Antiquity of Southeastern Europe Research Center, University of Warsaw, Krakowskie Przedmieście 32, 00-927 Warsaw, Poland; 5 Institute of Genetics and Biotechnology, University of Warsaw, Pawińskiego 5a, 02-106 Warsaw, Poland

**Keywords:** Microsatellite loci, Multiplex PCR, Atlantic sturgeon, European sturgeon

## Abstract

We have developed a multiplex assay covering 16 microsatellite loci, amplified in four polymerase chain reaction (PCR) assays, and loaded on the ABI DNA Analyzer in two separate panels. The assay was tested on 603 individuals originating from wild populations and hatchery stocks of Atlantic sturgeon. The assay was also tested on 12 individuals of European sturgeon and appeared to be almost equally useful. The multiplex assay designed in this study can be successfully applied in studies requiring high genetic resolution, such as relatedness analysis, selective breeding programs, and stock identification of Atlantic sturgeon.

**Electronic supplementary material:**

The online version of this article (doi:10.1007/s13353-014-0216-y) contains supplementary material, which is available to authorized users.

## Introduction

Atlantic (*Acipenser oxyrinchus*) and European (*A. sturio*) sturgeons belong to the Acipenseridae, considered one of the most endangered fish families (Pikitch et al. 2005; Ludwig 2006). Historically, Atlantic sturgeon inhabited the East Coast of North America, and Western and Central Europe, including the Baltic Sea, whereas the European sturgeon’s habitat was restricted to Western and Southern Europe (Ludwig et al. 2002 and 2009; Stankovic 2011; Chassaing et al. 2011a, 2013). During the last two centuries, the populations of Atlantic and European sturgeons have declined drastically due to river damming, habitat destruction, and overexploitation (Waldman and Wirgin 1998; Vecsei 2005; Ludwig 2006). At present, both species are endangered across the whole of their historical range; in the case of the European sturgeon, only a single population exists, in the Gironde–Garonne–Dordogne watershed (France) (Lassalle et al. 2011). The Atlantic sturgeon became extinct in the wild in Europe in 1996, when the last specimen was captured in the Baltic Sea (Paaver 1999). In Poland, a sturgeon restitution program was launched in 2004 (Kolman et al. 2011). Material from the St. John River has been used to build up broodstock in Poland and to repopulate local rivers (Kolman et al. 2011). These attempts at sturgeon restitution in the Baltic Sea have been followed by genetic monitoring using microsatellite loci to characterize and maintain genetic polymorphism within the established population.

Microsatellite loci are molecular markers commonly used in population genetics, especially in non-model organisms. The usefulness of these markers lies in their assumed neutrality and high level of polymorphism. In conservation genetics, microsatellite loci have been applied for the determination of management units (MUs), and for planning captive breeding programs and population management (DeSalle and Amato 2004). The number of loci needed to obtain reliable results depends upon the type of analysis required and the genetic variability of the species under study. For strain identification or relatedness analysis, the use of 15–20 markers to obtain sufficient statistical power is recommended (Blouin et al. 1996; Norris et al. 2000). Individual amplification and length analysis of so many loci is both time-consuming and expensive; therefore, the development and optimization of multiplex assays, enabling simultaneous amplification and analysis of several loci in a single polymerase chain reaction (PCR) assay, is becoming increasingly popular (Zhu et al. 2010; Olafsson et al. 2010; Ciofi et al. 2011). Carefully tested multiplexes allow for the direct comparison of populations studied in different laboratories and for building common databases of genotypes.

Various sets of microsatellite loci have already been employed to study wild and hatchery populations of Atlantic and European sturgeons (Ludwig et al. 2000, 2008; King et al. 2001; Wirgin et al. 2002; Henderson et al. 2004; Williot et al. 2007; Tiedemann et al. 2011; Saarinen et al. 2011; Moyer et al. 2012; Waldman et al. 2013; Wirgin et al. 2012). However, no standardized multiplex set is currently available, despite the utility of such an assay for the sturgeon conservation programs which have recently been started in several countries (Henderson et al. 2004; Gessner et al. 2011; Kirschbaum et al. 2011; Kolman et al. 2011). The aim of the present paper was to develop a microsatellite multiplex assay as a standard, cost-effective genotyping tool for the analysis of Atlantic sturgeon populations. The system was successfully tested on several wild and broodstock sturgeon populations, and we further show that the same approach could also be used for the analysis of the European sturgeon species.

## Materials and methods

### Sampling and DNA extraction

The material for this study consisted of 255 Atlantic sturgeon specimens from seven wild populations from the St. John, St. Lawrence, Kennebec, Hudson, Savannah, Shark, and Delaware Rivers, and 348 specimens from the broodstock kept in the Kuźniczki and Pieczarki Hatchery Stations in Poland and at the Leibniz Institute of Freshwater Ecology and Inland Fisheries in Germany (Supplementary Table 1). The broodstock population was composed of nine year classes, each being the progeny of artificially spawned wild individuals originating from the St. John River. As the number of spawners caught and used for reproduction was limited (usually around five to eight, annually), the year classes were mostly composed of siblings and half-siblings. Additionally, 12 wild specimens of European sturgeon from the Gironde population were analyzed (Supplementary Table 1). DNA was extracted from fin clips preserved in 70 % EtOH. DNA was isolated using proprietary kits [Sherlock AX Kit, Genomic Mini (A&A Biotechnology) or Wizard Kit (Promega)], according to manufacturers’ instructions. DNA was eluted with sterile water and stored at −20°C.

### Microsatellite selection and multiplex PCR optimization

Up to 2009, 38 microsatellite loci had been described from Atlantic sturgeon (May et al. 1997; King et al. 2001; Henderson-Arzapalo and King 2002). Of these, we chose 17 loci which had simple, long repeat motifs and which showed the highest polymorphism. These were assigned to four multiplex reactions based on their allele lengths (Supplementary Fig. 1). Before including different loci in the multiplex assays, their potential for primer dimer or hairpin formation was tested in silico, using FastPCR v.3.8.41 with default parameters (Kalendar et al. 2009). To avoid non-specific amplification, the forward primers for *Aox*D170 and *Ls*-62 were modified (Supplementary Table 2). The primer concentration was adjusted experimentally to give similar amounts of PCR products which were evaluated on the chromatogram reads. The final amplification reaction volume of 10 μl contained 50–200 ng of DNA, 1× MasterMix (Qiagen Multiplex Kit), and an appropriate concentration of primers, of which the 5′ end of each forward primer was fluorescently labeled (Supplementary Table 2). The PCR conditions were as follows: initial denaturation of 15 min at 95 °C, 35 cycles of 30 s at 94 °C, 30 s at 63 °C (mixes 1 and 2) or 61 °C (mixes 3 and 4), 30 s at 72 °C, and a final elongation of 30 min at 60 °C. Mixes were loaded onto two autosequencer panels (1 + 2 and 3 + 4). The length reading of PCR products was performed commercially (Oligo.pl, IBB, Warsaw, Poland) on ABI PRISM 377 and 310 automated sequencers with ROX 500 size standard (Applied Biosystems).

### Data analysis

Peak Scanner v.1.0 software (Applied Biosystems) was used to bin, score, and output the microsatellite data. Micro-Checker v.2.2.3 (Van Oosterhout et al. 2004) was applied to test for errors due to stuttering, null alleles (A_N_), and large allele dropouts. These analyses were performed for the whole dataset and each of the five most numerous wild populations, using 1,000 randomizations and 95 % confidence interval (CI). The Brookfield 1 (Brookfield 1996) equation was used for null allele frequency estimation. The number of alleles per locus (N_A_) and probability of identity (PI and PIsibs) were calculated using GenAlEx v.6.5 software (Paekal and Smouse 2001), while GENEPOP v.4.0.10 (Raymond and Rousset 1995) was used to calculate the mean null allele frequency (A_N_) using maximum likelihood based on the expectation–maximization (EM) algorithm. Observed heterozygosity (H_O_), expected heterozygosity (H_E_), and linkage disequilibrium between pairs of loci using 16,000 permutations were estimated with Arlequin v.3.5.1.2 (Excoffier and Lischer 2010). Linkage analysis was conducted for the five wild populations of sturgeons (excluding Savannah and Delaware, as their population sizes were considered too small). The average non-exclusion probabilities for parentage assignment (NE-1P, NE-2P, and NE-PP) and the polymorphic information Content (PIC) of the studied loci were calculated separately for the wild and hatchery populations using Cervus v.3.0 (Kalinowski et al. 2007).

## Results and discussion

### Multiplex establishment

The multiplex assay for 17 microsatellite loci was tested on a sample of 603 Atlantic sturgeons from both wild and hatchery populations (Tables 1 and 2). Using FastPCR, only the primers for loci *Aox*D161 and *Aox*D297 (mix 1) forward primer for locus *Ls*-68 (mix 4) could form dimers. However, the amplified products were, nevertheless, clearly distinguishable and easy to score. The obtained allele ranges for each locus of the 603 individuals belonging to the main distribution area of Atlantic sturgeon were non-overlapping (Supplementary Fig. 1). The total successful amplification rate of particular loci varied from 96.5 to 100 %, with the exception of locus *Ls*-62 (85.7 %) (Table 1). We subsequently decided to exclude this locus from the multiplex assay, as the low amplification yields were most probably caused by the presence of null alleles and stuttering, as suggested by the results obtained with the Micro-Checker software.

**Table 1:** Allele size ranges obtained for Atlantic and European sturgeons and gene diversity indices calculated only for Atlantic sturgeons (*N* = 603 individuals). N_A_—number of alleles; H_O_—observed heterozygosity; H_E_—expected heterozygosity; PIC—polymorphic information content; A_n_—null allele frequency calculated with GENEPOP and Micro-Checker

Locus	Size range of *A. oxyrinchus*	Size range of *A. sturio*	N_A_	Total percentage of amplifications	H_O_ total	H_E_ total	PIC total	A_n_ GENEPOP	A_n_ Micro-Checker
*Aox*C45	108–124	112–136	3	100	0.18	0.18	0.17	0	0
*Aox*D170	134–166	158–190	9	98.8	0.82	0.82	0.79	0.01	0
*Aox*D234	195–331	199–211	31	98.0	0.87	0.90	0.89	0.05	0.01
*Aox*45	109–169	103–145	19	99.7	0.75	0.74	0.71	0.01	−0.01
*Aox*D54	174–230	182–194	11	99.7	0.44	0.55	0.53	0.06	0.07
*Aox*D188	262–354	242–250	19	98.8	0.60	0.59	0.57	0.02	−0.01
*Aox*D161	126–158	162–170	9	99.8	0.71	0.74	0.69	0.03	0.02
*Aox*D297	185–353	169–173	36	97.0	0.64	0.82	0.80	0.10	0.10
*Ls*-68	120–172	144	11	98.7	0.64	0.77	0.73	0.08	0.07
*Aox*D241	180–272	148–184	24	99.5	0.89	0.90	0.89	0	0
*Aox*C55	114–130	118–142	5	100	0.23	0.23	0.21	0	0
*Aox*C27	150–174	150–166	6	100	0.55	0.51	0.40	0.02	−0.03
*Aox*D64	197–281	221	15	98.5	0.77	0.85	0.83	0.09	0.04
*Aox*D186	283–327	287	20	96.5	0.64	0.77	0.73	0.06	0.07
*Aox*D242	163–215	243–315	14	99.5	0.65	0.75	0.72	0.05	0.05
*Aox*C30	269–341	257	10	99.3	0.27	0.26	0.24	0	−0.01
Mean	−	−	15.2	99.0	0.60	0.65	0.62	−	−
*Ls*-62 (excluded)	71–83	75–87	4	85.7	0.27	0.37	0.33	0.09	0.07

**Table 2:** Genetic diversity of the two hatchery populations (a single year class and nine year classes) and of the seven wild populations from North American rivers of Atlantic sturgeon. N—number of individuals tested; N_A_—number of alleles; H_O_—observed heterozygosity; H_E_—expected heterozygosity; PIC—polymorphic information content; NE-1P, NE-2P, and NE-PP—average non-exclusion probabilities for the first and second parent and for the parent pair, respectively, calculated for samples of increasing genetic diversity; PI and PIsibs—probability of identity of unrelated and sibling individuals

	Hatchery (single year class)	Hatchery (9 year classes)	Wild populations
N	127	348	254
N_A_	3.6	9.0	14.8
H_O_	0.71	0.61	0.60
H_E_	0.55	0.60	0.68
PIC	0.46	0.56	0.66
NE-1P	0.04	0.005	0.0001
NE-2P	0.002	9.00 × 10^−5^	5.00 × 10^−7^
NE-PP	5.00 × 10^−5^	1.00 × 10^−7^	1.35 × 10^−11^
PI	5.82 × 10^−10^	3.48 × 10^−13^	6.02 × 10^−18^
PIsibs	8.00 × 10^−5^	1.00 × 10^−5^	1.20 × 10^−6^

### Linkage disequilibrium and null allele frequency estimation

Independently performed linkage analysis has shown that the microsatellite loci included into the multiplex assay are not linked with each other, as neither of the pair of loci was repeatedly significantly linked across all five examined populations. No evidence of large allele dropouts or false alleles was obtained. However, four loci (*Aox*D54, *Aox*D297, *Ls*-68, *Aox*D186) could exhibit stuttering according to Micro-Checker and four loci (*Aox*D186, *Aox*D54, *Aox*D297, *Ls*-68) could be biased with null allele presence, with the frequency exceeding 0.05 for both tests (Table 1). Analyses of the five wild populations separately have shown (according to the Brookfield 1 method) that only some of the loci could exhibit null alleles (data not shown), but not across all of the studied populations (*Aox*D186 in none, *Aox*D54 in one, *Ls*-68 in two, and *Aox*D297 in three populations). The highest A_n_ values, reaching 0.10, were obtained for *Aox*D297. However, as the most polymorphic locus, we consider this to be a valuable member of the multiplex assay. The A_n_ values obtained for other loci were usually close or equal to zero. The occurrence of null alleles in a large sample of individuals representing populations from a very broad geographic range is not a surprise considering the high mutation rates of the microsatellite loci ranging, on average, 5 × 10^−4^ per generation (Schlötterer 2004).

### Polymorphism and assignment power of the selected markers

The results presented in Table 1 indicate that all the loci included in the multiplex assay were polymorphic. The number of alleles varied from three (*Aox*C45) to 36 (*Aox*D297), and averaged 15.2 per locus, making this multiplex assay a valuable tool for population analysis. Observed heterozygosity also varied strongly between the loci, often departing from the mean value of 0.60. The PIC values, which depend on both allele frequency and heterozygosity, varied between 0.17 for *Aox*C45 and 0.89 for *Aox*D234 and *Aox*D241. As expected, all indices of genetic polymorphism were lower for the hatchery population than for wild fish, presumably as a result of using a limited number of spawners for reproduction (Table 2). Despite that, the PI values were still sufficiently high to distinguish even highly related individuals. The same was true also for the paternity analysis, where non-exclusion probabilities had very low values, minimizing the probability of errors of false parent identification. These results demonstrate that the designed multiplex assay could serve well for both stock identification and kinship analysis. Even when a single year class, containing the progeny of one female and two males, was analyzed (Table 2), the PI and non-exclusion probabilities were sufficiently low to assure proper individual and parental identification.

### Cross-species amplification in *A. sturio*

The multiplex assay was also tested on 12 individuals of European sturgeon (*A. sturio*). For five loci, the optimal annealing temperature had to be lowered to 58 °C (*Aox*D188, *Aox*C27 56 °C (*Aox*C45), and 54 °C (*Aox*D242) for successful amplification, but even at these temperatures, the amplification of *Aox*C45 and *Aox*D242 remained problematic. Four loci seemed to be monomorphic (Table 1), although analysis of a larger sample may reveal more alleles for these loci, as shown previously for *Ls*-68 (Chassaing et al. 2011b). The low variability of European sturgeon is not surprising, because only a single, very small, and probably bottlenecked population of species remains (Williot et al. 2007). For five loci (*Aox*D161, *Aox*D188, *Aox*D297, *Aox*D242, and *Aox*C30), the allele range sizes of Atlantic and European sturgeons did not overlap. These loci together could serve as a powerful tool for species or hybrid identification. Chassaing et al. (2011b) has already suggested the use of *Aox*D161 as a diagnostic marker distinguishing Atlantic and European sturgeons. If the multiplex assay described here is used to analyze European sturgeon populations, we suggest the use of a fourth fluorescent dye to label microsatellite loci *Aox*D161, *Aox*D242, and *Ls*-68, as the allele size ranges of these loci overlap with those of some others.

## Conclusions

The described multiplex assay, covering 16 microsatellite loci, amplified in four polymerase chain reaction (PCR) assays and loaded on the ABI DNA analyzer in two separate panels, can be successfully applied in the genetic analysis of populations and relatedness assignment in captive breeding programs of Atlantic sturgeon. The optimized microsatellite set may also be used for European sturgeon; however, for optimal results in this case, we recommend the addition of loci designed specifically for this species (Tiedemann et al. 2011). Five loci were identified which can be used for the detection of introgression between the two sturgeon species. The designed multiplex assay has already been applied in practice and will be used for creating a pedigree book and optimal cross design for the broodstock of Atlantic sturgeon during its reintroduction in Poland (Stankovic 2011).

## Electronic supplementary material

Below are the links to the electronic supplementary material.

Supplementary Table 1 (PDF 165 kb)

Supplementary Table 2 (PDF 242 kb)

Supplementary Fig. 1 Multiplex PCR loading panels showing allele size range and colors assigned to microsatellite primers labeled with 6-FAM, Hex, and TAMRA for the 603 analyzed specimens of Atlantic sturgeon. (PDF 214 kb)
