# Supplementary material for: Microsatellite multiplex assay for the analysis of Atlantic sturgeon populations
Source: J Appl Genet. 2014 May 4;55(4):505–10. doi: 10.1007/s13353-014-0216-y (PMC4185099; doi:10.1007/s13353-014-0216-y)
Supplement: Supplementary file 1 — (PDF 165 kb) [file 13353_2014_216_MOESM1_ESM.pdf]

Microsatellite multiplex assay for analysis of Atlantic sturgeon populations

Panagiotopoulou H[1*], Popovic D[2], Zalewska K[3], Weglenski P[1,2] and Stankovic A[1,3,4]

[1] Institute of Biochemistry and Biophysics, Polish Academy of Science, Pawińskiego 5a, 02-106 Warsaw, Poland

[2]Centre of New Technologies (CeNT),University of Warsaw, ul. Żwirki i Wigury 93, 02-089 Warsaw

[3] Faculty of Biology, University of Warsaw, Institute of Genetics and Biotechnology;

Pawińskiego 5a, 02-106 Warsaw, Poland

[4] The Antiquity of Southeastern Europe Research Center, University of Warsaw, Krakowskie Przedmieście 32, 00-927 Warsaw, Poland

*Corresponding author:

Panagiotopoulou Hanna

Institute of Genetics and Biotechnology;

Pawińskiego 5a, 02-106 Warsaw, Poland

Phone: +48 22 5923233

Fax: +48 226584176

hpana@wp.pl

Supplementary Table 1. Providers, number and geographical origin of sturgeon samples analyzed in the presented work.

Population origin (species)	Sample number	Provider of the biological material for genetic analyses
Saint Lawrence River, Canada<br>(*A. oxyrinchus*)	54	T.L. King and B. Lubinski, USGS Leetown Science Center, USA
Saint John River, Canada<br>(*A. oxyrinchus*)	94	R. Kolman and M. Szczepkowski,Inland Fisheries Institute, Poland<br>J. Gessner, Leibniz-Institute of Freshwater Ecology and Inland Fisheries in Germany
Kennebec River, USA<br>(*A. oxyrinchus*)	60	J. Bartlett and G. Wipplehauser, Maine Department of Marine Resources, USA<br>I. Wirgin, NYU Langone Medical Center, USA
Hudson River, USA<br>(*A. oxyrinchus*)	20	T.L. King and B. Lubinski, USGS Leetown Science Center, USA
Savannah River, USA<br>(*A. oxyrinchus*)	5	T.L. King and B. Lubinski, USGS Leetown Science Center, USA
Delaware River, USA<br>(*A. oxyrinchus*)	9	B. May, Department of Animal Science, University of California, Davis, USA<br>A. Ludwig, IZW-Berlin, Germany
Shark River, USA<br>(*A. oxyrinchus*)	13	B. May, Department of Animal Science, University of California, Davis, USA<br>A. Ludwig, IZW-Berlin, Germany
9 year-classes of broodstock (Saint John River origin), Polish and German hatchery stations (*A. oxyrinchus*)	348	R. Kolman and M. Szczepkowski, Inland Fisheries Institute, Poland<br>M. Raczkowski, Kuźniczki Hatchery Station, Poland<br>J. Gessner from Leibniz-Institute of Freshwater Ecology and Inland Fisheries, Germany
Gironde, France<br>(*A. sturio*)	12	E. Rochard, CEMAGREF Bordeaux, Unité de recherche : Ecosystèmes estuariens et poissons migrateurs amphihalins France
