# Supplementary material for: Microsatellite multiplex assay for the analysis of Atlantic sturgeon populations
Source: J Appl Genet. 2014 May 4;55(4):505–10. doi: 10.1007/s13353-014-0216-y (PMC4185099; doi:10.1007/s13353-014-0216-y)
Supplement: Supplementary file 2 — (PDF 242 kb) [file 13353_2014_216_MOESM2_ESM.pdf]

Microsatellite multiplex assay for analysis of Atlantic sturgeon populations

Panagiotopoulou H[1*], Popovic D[2], Zalewska K[3], Weglenski P[1,2] and Stankovic A[1,3,4]

[1] Institute of Biochemistry and Biophysics, Polish Academy of Science, Pawińskiego 5a, 02-106 Warsaw, Poland

[2]Centre of New Technologies (CeNT),University of Warsaw, ul. Żwirki i Wigury 93, 02-089 Warsaw

[3] Faculty of Biology, University of Warsaw, Institute of Genetics and Biotechnology;

Pawińskiego 5a, 02-106 Warsaw, Poland

[4] The Antiquity of Southeastern Europe Research Center, University of Warsaw, Krakowskie Przedmieście 32, 00-927 Warsaw, Poland

*Corresponding author:

Panagiotopoulou Hanna

Institute of Genetics and Biotechnology;

Pawińskiego 5a, 02-106 Warsaw, Poland

Phone: +48 22 5923233

Fax: +48 226584176

hpana@wp.pl

Supplementary Table 2. Symbols of microsatellite loci, their accession numbers, primer sequences, repeat motifs and dyes employed in the PCR multiplexes. Attribution of the loci to four PCR mixes (Mix) and the optimized primer concentrations (µM) are also given. The

numbers at the loci symbols refer to the following papers: a -Henderson-Arzapalo and King 2002; b- May et al., 1997; c - King et al., 2001.

Locus	GenBank Acc. No		Primer sequence	Repeat motif	µM	Dye	Mix
*Aox*C45[a]	AY093632	For	GCGACCCTGTAAAGGAGTAAG	$(GGAT)_8$	0.1	6-FAM	
		Rev	TATTAAACTTGGACCGGTTAGC				
*Aox*D54[a]	AY093636	For	GAGAACAACGTTTTACTGCAAAC	$(TAGA)_{18}$	0.1	HEX	
		Rev	GATCATAACTAAAGCTGGCAGG				
*Aox*D161[a]	AY093639	For	GTTTGAAATGATTGAGAAAATGC	$(CTAT)_{15}$	0.1	TAMRA	1
		Rev	TGAGACAGACACTCTAGTTAAACAGC				
*Aox*D297[a]	AY093648	For	TGCTTGTATTTTCTGTTTTAGCC	$(TAGA)_{20}$	0.2	TAMRA	
		Rev	CCACACTAGTGCATCCAGCTC				
*Aox*45[c]	AF067813	For	TTGTCCAATAGTTTCCAACGC	$(AAT)_{20}$	0.1	HEX	
		Rev	TGTGCTCCTGCTTTTACTGTC				
*Aox*D170[a]	AY093641	For	GAACCATTTTATTGACATTCGACGG	$(TAGA)_{16}$	0.1	6-FAM	
		Rev	CCCTGTCTCACGTACATTTTATG				
*Aox*D188[a]	AY093644	For	TGAAGTCATTGGTGATGTGTATG	$(GTAT)_5(CTAT)_4$CTGT $(CTAT)_2$CTGT$(CTAT)_{15}$	0.3	HEX	2
		Rev	ATGGAAATGTTTTATGGTAATGTG				
*Aox*D234[a]	AY093645	For	AACTGGCTTTGTGATTGATCC	$(TAGA)_{17}$	0.1	6-FAM	
		Rev	TGAAGCAAAGGGTATTATTTGAG				
*Aox*D64[a]	AY093637	For	TTTGTGTAGGGAAATACCCTTG	$(TAGA)_{16}$	0.2	HEX	
		Rev	TGAGTGCAGCCCTACTGCTC				
*Aox*D242[a]	AY093647	For	ATACATCACCCAGGACAAGTTC	$(CTAT)_{18}$	0.1	TAMRA	
		Rev	AACAATGTTGCAATTCTGTGG				
*Aox*C27[a]	AY093629	For	CCACGACCCTCTATAGGATTAAG	$(GGAT)_7$	0.2	HEX	3
		Rev	TATAGGAATTATTTTGCCCCAG				
*Aox*C55[a]	AY093633	For	GCAAGGTGTATTAAACTGGACC	$(CATC)_8(CATT)_2$AT CATT	0.1	HEX	
		Rev	CGACCCTGTAAAGGAGTAAGC				
*Aox*C30[a]	AY093631	For	TCTCTAAATTGCGCCTTAGTTG	$(GGAT)_8(AGAT)_2$	0.1	TAMRA	
		Rev	TTTTGGAATTGCTGTCTAACTG				
*Aox*D186[a]	AY093643	For	TGATCCTTGTGACATAAACAGAAC	$(CA)_8(TA)_2$TT$(TA)_9$	0.1	HEX	4

		Rev	CTGAGAAACACTTGCTTGTTTG	$(GGAT)_7GGA(TAGA)_{19}$		
*Aox*D241[a]	AY093646	For	TGTTCACAATATAGTCTTCCAGGTC	$(TAGA)_{36}$	0.05	6-FAM
		Rev	CACAACAAATCAAAACAGAAGC			
Ls-68 [b] (Afu-68)	U72739	For	TTATTGCATGGTGTAGCTAAAC	$(GATA)_{13}$	0.4	6-FAM
		Rev	AGCCCAACACAGACAATATC			
Ls-62[b] (Afu-62)	U72738	For	GATCAGGAGGGCAGAG	$(GACA)_7$	0.3	6-FAM
		Rev	CCCTGGATTTGAATTAACAG			
