# Supplementary material for: Microsatellite multiplex assay for the analysis of Atlantic sturgeon populations
Source: J Appl Genet. 2014 May 4;55(4):505–10. doi: 10.1007/s13353-014-0216-y (PMC4185099; doi:10.1007/s13353-014-0216-y)
Supplement: Supplementary file 3 — Multiplex PCR loading panels showing allele size range and colors assigned to microsatellite primers labeled with 6-FAM, Hex, and TAMRA for the 603 analyzed specimens of Atlantic sturgeon. (PDF 214 kb) [file 13353_2014_216_MOESM3_ESM.pdf]

Microsatellite multiplex assay for analysis of Atlantic sturgeon populations

Panagiotopoulou H[1*], Popovic D[2], Zalewska K[3], Weglenski P[1,2] and Stankovic A[1,3,4]

[1] Institute of Biochemistry and Biophysics, Polish Academy of Science, Pawińskiego 5a, 02-106 Warsaw, Poland

[2]Centre of New Technologies (CeNT),University of Warsaw, ul. Żwirki i Wigury 93, 02-089 Warsaw

[3] Faculty of Biology, University of Warsaw, Institute of Genetics and Biotechnology;

Pawińskiego 5a, 02-106 Warsaw, Poland

[4] The Antiquity of Southeastern Europe Research Center, University of Warsaw, Krakowskie Przedmieście 32, 00-927 Warsaw, Poland

*Corresponding author:

Panagiotopoulou Hanna

Institute of Genetics and Biotechnology;

Pawińskiego 5a, 02-106 Warsaw, Poland

Phone: +48 22 5923233

Fax: +48 226584176

hpana@wp.pl

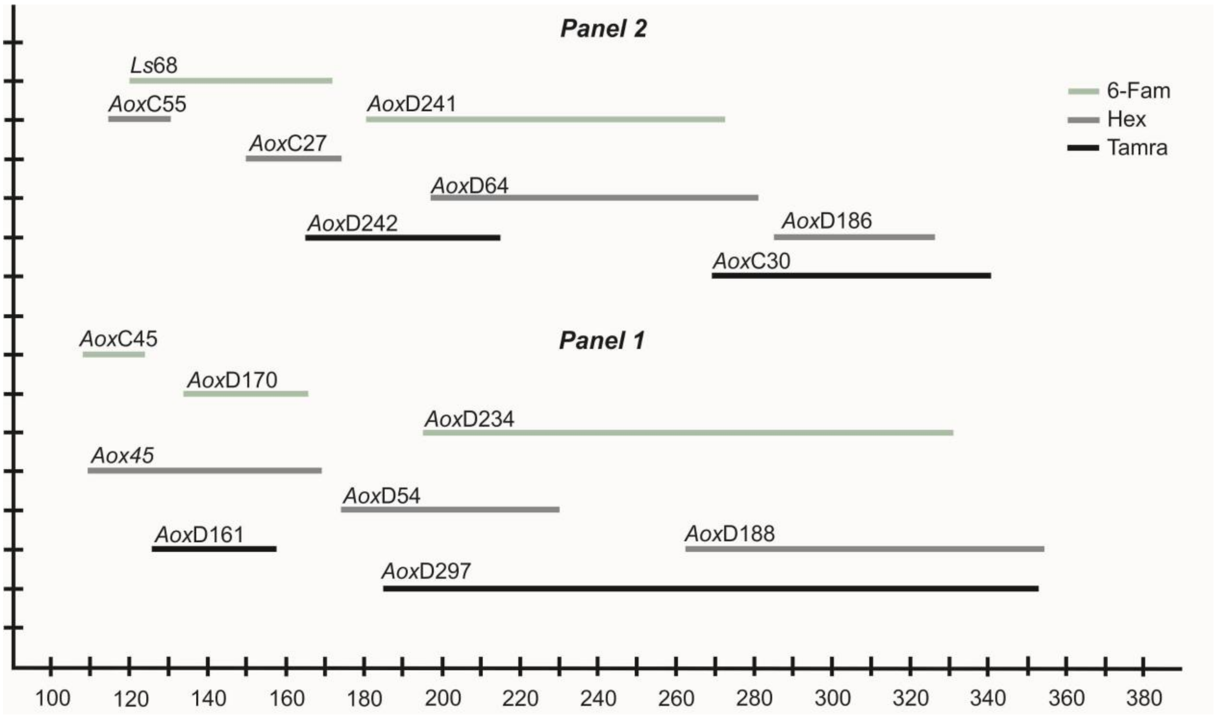
Panel 2
Ls68
AoxC55
AoxD241
AoxC27
AoxD64
AoxD242
AoxD186
AoxC30
Panel 1
AoxC45
AoxD170
AoxD234
Aox45
AoxD54
AoxD161
AoxD188
AoxD297
6-Fam
Hex
Tamra
100 120 140 160 180 200 220 240 260 280 300 320 340 360 380
